# A new strategy for cardiac protection

**DOI:** 10.7554/eLife.93239

**Published:** 2023-11-02

**Authors:** Esmaa Bouhamida, Hina W Chaudhry

**Affiliations:** 1 Cardiac Regenerative Medicine Laboratory and the Department of Cardiology, Icahn School of Medicine at Mount Sinai New York United States

**Keywords:** sirtuins, cardiac hypertrophy, heart failure, Mouse

## Abstract

It may be possible to treat cardiac hypertrophy and injury by using drugs that inhibit a protein called SIRT2.

**Related research article** Yang X, Chang HC, Tatekoshi Y, Mahmoodzadeh A, Balibegloo M, Najafi Z, Wu R, Chen C, Sato T, Shapiro J, Ardehali H. 2023. SIRT2 inhibition protects against cardiac hypertrophy and ischemic injury. *eLife* **12**:e85571. doi: 10.7554/eLife.85571.

Cardiac hypertrophy is a complicated medical condition that occurs when muscle cells in the heart increase in size in response to pressure overload as they lose the ability to proliferate after birth due to exit from the cell cycle (Chaudhry et al., 2004; Cheng et al., 2007; Mohamed et al., 2018). Cardiac hypertrophy can sometimes arise through physiological adaptation to exercise or pregnancy. However, it can also be pathological – when, for example, it is caused by long-term hypertension – and this can lead to ischemic heart disease, valvular disorders, and heart failure (Frey et al., 2004; Bouhamida et al., 2023; Morciano et al., 2021). Unfortunately, treatment options are limited, and there are relatively few therapies that directly target heart function and remodeling. There is a need, therefore, to better understand the molecular mechanisms that trigger cardiac hypertrophy, so that researchers can develop new therapeutic approaches that can prevent or slow down the development of this condition and the heart diseases it causes.

Now, in eLife, Hossein Ardehali and colleagues at the Northwestern University School of Medicine – including Xiaoyan Yang, Hsiang-Chun Chang and Yuki Tatekoshi as joint first authors – report the results of experiments on mice that shed light on the molecular mechanisms involved in cardiac hypertrophy (Yang et al., 2023). In particular, they focus on the regulation of a transcription factor known as NRF2 by a protein called SIRT2, which is a member of the Sirtuin family of signaling proteins.

Sirtuin proteins are involved in a wide range of cellular processes, such as aging, cell death, inflammation, and mitochondrial biogenesis (Baur et al., 2012; Preyat and Leo, 2013; Pinton et al., 2007). Recent studies have also suggested that SIRT2 has a role in cardiac hypertrophy (Tang et al., 2017) and heart failure (Sarikhani et al., 2018), although the precise nature of this role has remained unclear. Yang et al. employed a range of different molecular biology and immunogenetics methods to verify gene and protein expression levels, and performed a range of in vitro and in vivo studies, including experiments on mice that lacked the genes for SIRT2 and NRF2.

The researchers showed that SIRT2 was expressed in the heart of wild-type mice, and that the expression of SIRT2 was higher in mice that had been subjected to trans-aortic constriction. They also found increased levels of SIRT2 in hearts explanted from patients with end-stage heart failure due to dilated cardiomyopathy, and in hearts from patients with ischemic cardiomyopathy. Moreover, Yang et al. found that mice deficient in the gene for SIRT2 displayed improved cardiac function in response to pressure overload and ischemia/reperfusion injury. These mice also showed reduced levels of various markers for heart failure following cardiac injury: further, this effect was not gender specific.

Consistent with these results, when short interfering RNA was used to downregulate Sirt2 mRNA in in vitro experiments on neonatal cardiac cells taken from rats, the researchers observed a protective effect against stress-induced cell death. Overall, the results suggest that SIRT2 has a detrimental effect when the heart has been subjected to pressure overload or ischemia/reperfusion injury, and that deletion of the gene for SIRT2 protects against cardiac hypertrophy and ischemic injury.

Yang et al. then went on to identify one of the mechanisms by which SIRT2 deficiency helps protect the heart. This mechanism involved NRF2, a transcription factor that activates genes that code for various antioxidative enzymes and other proteins that protect cells against harmful agents. The researchers found that a lack of SIRT2 triggers the activation of this transcription factor, and increases the transfer of NRF2 from the cytoplasm to the cell nucleus, which leads to higher levels of antioxidants being expressed in the nucleus (Figure 1). Moreover, deletion of the gene for NRF2 can reverse the protection provided by the deletion of the gene for SIRT2. Finally, the researchers went on to show that the in vivo administration of AGK2 – a drug that selectively inhibits SIRT2 – improved cardiac remodeling and protected the heart against cardiac hypertrophy.

**Figure 1.**
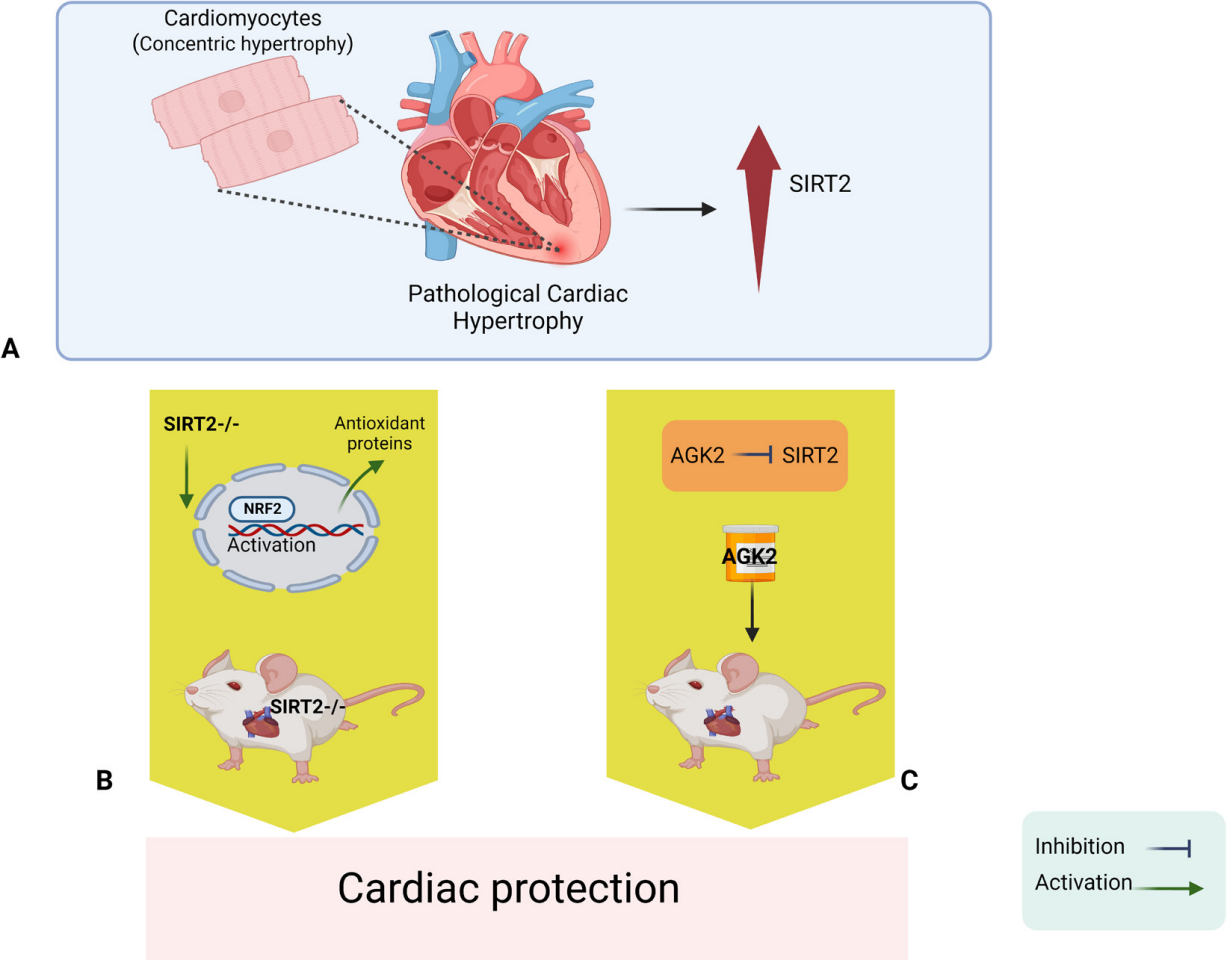
Sirt2 deletion protects the heart against cardiac hypertrophy and injury. (**A**) Pathological cardiac hypertrophy is associated with increased levels of a signaling protein called SIRT2. (**B**) Deleting the gene for SIRT2 in mice leads to higher levels of a transcription factor called NRF2 in the nucleus; NRF2 then activates various antioxidant proteins that protect the heart against cardiac hypertrophy and ischemic injury. (**C**). In vivo administration of a drug called AGK2 also protects the heart because it inhibits SIRT2. NRF2: nuclear factor erythroid 2-related factor 2; SIRT2: sirtuin 2.

Image credit: Figure created by EB using BioRender.

The cardioprotective effect of SIRT2 has been the subject of debate and controversy, and the findings by Yang et al. are inconsistent with some previous reports. For example, one study demonstrated that mice with SIRT2 deficiency exhibit enhanced pathological cardiac hypertrophy (Sarikhani et al., 2018), while another reported that SIRT2 deletion induced aging-dependent and angiotensin II-mediated pathological cardiac hypertrophy (Tang et al., 2017). However, Yang et al. demonstrated the deletion of SIRT2 has a cardioprotective effect regardless of whether SIRT2 is deleted from all cells or specifically from those of the heart. Moreover, they provided a new molecular mechanism for the protective effect of SIRT2 deletion, and also identified a potential therapeutic approach through the selective inhibition of SIRT2.

Possible explanations for the differences between previous studies and the latest work could be the genetic background of the mice, the different approaches used to target the gene for SIRT2, or the different methods to mimic cardiac hypertrophy.
